# Food Crops (Poaceae, Solanaceae, and Fabaceae) with Anti-Cancer Nutraceutical Prospects: A Review of Plant Extracts

**DOI:** 10.3390/molecules31122126

**Published:** 2026-06-16

**Authors:** Edna C. Blanco-Torres, Gildardo Rivera, Timoteo Delgado-Maldonado, Eyra Ortiz-Pérez, Alma D. Paz-González, Ana Verónica Martínez-Vázquez, Erick de Jesús de Luna-Santillana, Jessica L. Ortega-Balleza, Lenci K. Vázquez-Jiménez

**Affiliations:** 1 Laboratorio de Biotecnología Farmacéutica, Centro de Biotecnología Genómica, Instituto Politécnico Nacional, Reynosa 88710, Mexico; eblancot2400@alumno.ipn.mx (E.C.B.-T.); giriveras@ipn.mx (G.R.); titi_999@live.com (T.D.-M.); eortizp@ipn.mx (E.O.-P.); apazg@ipn.mx (A.D.P.-G.); avmartinez@ipn.mx (A.V.M.-V.); jessica_ortega7@hotmail.com (J.L.O.-B.); 2 Laboratorio de Medicina de Conservación, Centro de Biotecnología Genómica, Instituto Politécnico Nacional, Reynosa 88710, Mexico; edeluna@ipn.mx

**Keywords:** cancer, natural products, nutraceuticals, anticancer drugs, cancer cells

## Abstract

Cancer is one of the leading causes of death worldwide and is characterized by the uncontrolled proliferation of cells. The ongoing study of this disease has been vital for designing more effective therapeutic strategies for treating various types of cancer. In this regard, natural chemistry as a cancer treatment represents a promising alternative for redefining therapies against this disease. Natural products for treating cancer, classified as nutraceuticals, are emerging as highly interesting alternatives due to their ability to participate in different cellular pathways and their low toxicity. This study aims to provide an overview of the potential of nutraceuticals derived from edible plants in the prevention and complementary treatment of cancer, including their reported bioactive compounds and mechanisms of action.

## 1. Introduction

Cancer comprises a group of diseases characterized by the uncontrolled proliferation of abnormal cells capable of invading adjacent tissues and spreading to other organs through metastatic processes. These cellular alterations arise because of genetic and epigenetic changes that affect the normal mechanisms of cell cycle regulation, apoptosis, and DNA repair [1]. Due to its multifactorial nature and the complexity of its molecular mechanisms, cancer continues to represent one of the major challenges to public health worldwide.

According to global estimates, cancer remains among the leading causes of death in the world population. Recent data indicate that in 2020, approximately 19.3 million new cases and nearly 10 million deaths associated with this disease were recorded [2]. Furthermore, epidemiological projections suggest that by 2040 the number of new cases could reach 28.4 million, representing an increase of approximately 47% compared to current figures [2]. This increase is related to various factors, including population aging, changes in lifestyles, and exposure to environmental and behavioral factors associated with disease development [3].

Among the main risk factors involved in carcinogenesis are tobacco and alcohol consumption, exposure to carcinogenic substances, radiation, obesity, chronic infections, genetic predisposition, persistent inflammation, and certain dietary patterns. Collectively, these factors can induce molecular alterations that promote malignant cell transformation and tumor progression [4].

Although conventional cancer treatments, such as chemotherapy and radiotherapy, have proven effective in various types of cancer, their use is often associated with significant adverse effects, including systemic toxicity, damage to healthy tissue, and the development of therapeutic resistance [5]. In recent years, newer strategies such as targeted therapies and immunotherapy have shown significant progress; however, they still have limitations related to their efficacy in certain types of cancer, unexpected side effects, and high treatment costs [6].

In this context, the search for new anticancer agents of natural origin has gained increasing interest in biomedical research. Several natural products have demonstrated relevant pharmacological properties, particularly plant-derived compounds rich in polyphenols, flavonoids, carotenoids, alkaloids, and terpenoids. During the early stages of carcinogenesis, these compounds can contribute to the prevention of cell damage by reducing oxidative stress, modulating chronic inflammatory processes, and regulating enzymes involved in carcinogen detoxification [7,8]. Furthermore, in established tumor cells, they have been shown to inhibit cell proliferation, induce apoptosis, and modulate signaling pathways associated with tumor survival, including PI3K/Akt, MAPK, NF-κB, and STAT3. In addition, some phytochemicals have demonstrated the ability to inhibit angiogenesis and tumor metastasis, and to increase the sensitivity of cancer cells to chemotherapy and radiotherapy, suggesting their potential as adjuvant agents in cancer treatment [9,10].

Among the most promising sources of these plant-based foods are those belonging to widely consumed botanical families such as Poaceae, Solanaceae, and Fabaceae, which have nutritional relevance, a broad global distribution, and growing scientific evidence regarding their anticancer potential. These families include some of the most consumed edible plants globally and represent an important source of bioactive compounds with nutraceutical and pharmacological properties [11,12].

The Poaceae family includes cereals that are fundamental to the human diet, such as rice, wheat, oats, barley, and maize, which contain phenolic compounds, flavonoids, anthocyanins, phytosterols, and tocopherols associated with antioxidant, anti-inflammatory, and antiproliferative activities [13,14,15].

The Solanaceae family includes widely consumed vegetables such as tomatoes, potatoes, eggplants, and chili peppers, characterized by their distinctive metabolites, including carotenoids, steroidal alkaloids, and capsaicinoids. Compounds like lycopene, solasonine, solamargine, and capsaicin have shown the ability to induce apoptosis, modulate the cell cycle, and regulate cancer-related signaling pathways [16,17,18].

As for Fabaceae, this family represents one of the main plant sources of protein and phenolic compounds in the human diet. Legumes such as lentils, beans, peas, chickpeas, and soybeans contain flavonoids, isoflavones, bioactive polysaccharides, and other metabolites with chemopreventive, antioxidant, and immunomodulatory properties [19,20]. Taken together, these three botanical families can be considered representative models due to their nutritional importance, phytochemical diversity, and experimental evidence related to anticancer mechanisms. In this sense, the study of these crops as potential sources of nutraceuticals represents a relevant strategy for the development of preventive or adjuvant alternatives in cancer treatment.

Therefore, this review analyzes the available scientific evidence on the anticancer potential of edible crops belonging to these botanical families, integrating results from in vitro studies that demonstrate their possible role in the prevention and control of various types of cancer.

## 2. Search Methodology

The research articles included in this review focused on in vitro studies of extracts from the most representative plants of the Poaceae, Solanaceae, and Fabaceae families. Cytotoxicity values were described in the analyzed studies, and in those that allowed it, anticancer mechanisms of action, selectivity, and phytochemical analyses were also described. Only research articles published in peer-reviewed journals were selected.

This literature review was conducted using the PubMed and Google Scholar electronic databases. The medical subject headings used were the terms “Poaceae”, “Solanaceae”, “Fabaceae”, “extract [common name of the plant]”, “extract [scientific name of the plant]”, “[scientific name of the plant] anticancer activity,” and “[common name of the plant] anticancer activity”. The same strategy was used for both databases, with the appropriate adaptations. The data presented were published between 2010 and 2025.

## 3. Poaceae Family

Poaceae are commonly known as cereals, and their cultivation accounts for approximately 70% of the planet’s arable land [21]. This family includes the four most important crops in the world, sugarcane, wheat, rice, and maize, representing half of global production in 2020 [22,23]. These crops form the base of the food pyramid because their nutritional value has a significant influence on health [22,24].

### 3.1. Rice

Rice (*Oryza sativa* L.) is a grain crop and a staple food for most of the world’s population, and an important agricultural product globally, with approximately 800 million tons produced in 2020 [23]. Furthermore, rice has shown pharmacological properties that could help prevent cancer formation [25,26,27]. Rice callus suspension cultures are among the most analyzed (Table 1). These are generated by cultivating calluses in a plant growth medium and are an alternative that can be used for the mass production of specific compounds through plant cell cultures in bioreactors [28,29,30]. Table 1 shows that the callus culture suspension exhibited inhibition against RXF-393 renal cancer cells (82%) and SW620 colon cancer cells (71%) at 1 mg/mL. Furthermore, it showed apparent favorable selectivity, as it did not exhibit toxicity in human MRC-5 lung fibroblasts. The authors attributed this activity to cellular structural alterations (determined by microscopic analysis) likely caused by synergistic effects among various secondary metabolites present in the plant cell culture [31].

Rice bran is another important byproduct of the rice milling industry, with a global potential of 29.3 million tons per year [37]. Other byproducts left in the fields after rice stems and leaves, which in recent years have been shown to have nutritional content and pharmacological potential [38,39]. Rice bran extracts and fermentation products have been described as playing important roles in mediating inflammation, the cell cycle, cell apoptosis, and cancer prevention [40,41,42]. Table 1 shows that extracts derived from bran, leaves, and cultures exhibit moderate to high cytotoxic effects. The bran extract showed moderate activity against HepG2, SW620, KATO-III, BT474 and ChaGo-K-1, with ChaGo-K-1 being particularly prominent with an IC_50_ of 25.6 μg/mL. Phytochemical analysis revealed high concentrations of phenols, flavonoids, anthocyanins and cyanidin-3-*O*-glucoside (determined by colorimetric methods), metabolites associated with antioxidant and anticancer properties (determined by DPPH, ABTS and FRAP assays) [32].

Ethanolic and methanolic extracts of rice bran showed the ability to induce apoptosis, inhibit angiogenesis, and modulate the immune response by decreasing VEGF and regulating cytokines (measured by ELISA, lymphocyte proliferation assays, pinocytic activity, phagocytic activity, and Th1/Th2 cytokine detection). These effects were associated with the presence of *p*-coumaric acid, ferulic acid, salicylic acid, saponarin, and the bioactive lipid FAHFA, identified by LC-MS. Interestingly, although the n-hexane extract showed lower concentrations of these metabolites, it exhibited better activity against some cell lines, such as MCF-7 and T47D, suggesting that lipophilic compounds may play an important role in cytotoxic activity. Furthermore, incorporating rice bran into the diet of mice resulted in a 20% regression in tumor development and growth [33]. The phenolic subfraction of leaves exhibited moderate activity against MCF-7, Caco-2, and HepG2, with IC_50_ values between 74 and 95 μg/mL. The mechanism of action involved apoptosis mediated by mitochondrial pathways and death receptors, accompanied by suppression of the PI3K/Akt and Erk pathways. These mechanisms were confirmed by flow cytometry, Annexin V, and Western blot. Furthermore, the extract showed high antioxidant capacity in DPPH and ABTS assays, suggesting that redox regulation may also contribute to the antiproliferative effect. HPLC-DAD-MS/MS (High-performance liquid chromatography-diode array detector-tandem mass spectrometry) analysis identified various flavonoids and phenolic compounds, including luteolin, luteolin-7-*O*-glucoside, luteolin-8-*C*-glucoside, quercetin-3-galactoside, and kaempferol-7-*O*-glucoside. These metabolites are recognized for modulating apoptosis, oxidative stress, and cell signaling related to tumor proliferation [27].

Less polar extracts (e.g., hexane or dichloromethane) showed anti-invasive activity against MDA-MB-231 and HT1080 related to the inhibition of metalloproteinases and collagenases (determined by Gelatin zymography assay and Collagenase activity assay), suggesting a possible antimetastatic effect. Furthermore, high concentrations of γ-oryzanol and γ-tocotrienol, compounds widely associated with antioxidant and anticancer properties, were identified (determined by HPLC) [35]. Water extracted from germinated brown rice showed low cytotoxic activity against A549, HepG2, and KKU-213, with IC_50_ values greater than 650 μg/mL. However, a reduction in S-phase cell progression was observed, suggesting a possible moderate antiproliferative effect. ^1^H-NMR analysis identified amino acids, sugars, phenolic compounds, and nitrogenous metabolites [34]. The ethanolic extract showed greater activity against Caco-2 compared to the aqueous extract, inducing DNA fragmentation through intrinsic and extrinsic apoptotic pathways as determined by a TUNEL assay and qRT-PCR (Quantitative Reverse Transcription Polymerase Chain Reaction). Although the aqueous extract had a higher total content of phenolic compounds, the ethanolic extract showed greater antioxidant activity (using the DPPH, ABTS, and FRAP assays) and cytotoxicity, probably due to more efficient extraction of specific bioactive compounds such as anthocyanins and lipophilic metabolites (determined by the differential pH and Folin–Ciocalteu colorimetry method) [36].

On the other hand, Punvittayagul et al. [43] investigated the effects of a methanolic extract of purple rice hulls on chemically induced carcinogenesis in rats. They observed that the purple rice hull extract showed no toxicity, clastogenicity, or carcinogenicity in rats (LD_50_ < 2000 mg kg^−1^ body weight). Additionally, oral administration of 300 or 1000 mg kg^−1^ body weight of the purple rice hull extract for 28 days significantly decreased the number of micronucleated hepatocytes in diethyl nitrosamine-initiated rats. The inhibitory mechanisms were associated with the reduction of cytochrome P450 2E1 expression and the induction of certain detoxifying enzymes in the liver. It significantly inhibited the formation of foci positive for hepatic glutathione S_2_ transferase induced by diethyl nitrosamine and 1,2-dimethylhydrazine by suppressing hepatocyte proliferation and inducing apoptosis.

Recently, Sasaki et al. [44] developed nanoparticles from Koshihikari rice bran and examined their anticancer activity and effect on peritoneal dissemination of murine colon adenocarcinoma cells in mice with tumors. The rice bran nanoparticles significantly reduced the number of colon26 cells, murine melanoma cells (B16-BL6), and human cervical adenocarcinoma cells (HeLa) in a concentration-dependent manner. The rice bran nanoparticles showed no significant cytotoxicity against non-cancer cells, including the MDCK canine kidney cell line and the HaCaT human keratinocyte cell line. The rice bran nanoparticles induced cell cycle arrest and apoptosis, and reduced the expression of proliferative proteins, including β-catenin and cyclin D1. Intraperitoneal injections of nanoparticles derived from rice bran in mice with peritoneal dissemination of colon26 cells significantly suppressed tumor growth without significant adverse effects.

### 3.2. Corn

Corn (*Zea mays* L.) is the most widely cultivated grain crop and one of the most important food crops, being one of the primary cereal grains produced globally, with an annual production exceeding 1.2 billion tons [45,46]. Although it is one of the most widely produced cereals, few studies have been published on its potential anticancer properties.

Balasubramanian et al. [47] investigated the ability of different extracts (aqueous, methanolic, and chloroform) of maize leaves to influence the process of hydrogen peroxide-induced apoptosis in Hep2 cells (squamous cell carcinoma). Treatment with hydrogen peroxide causes cytotoxicity in cancer cells. Administration of the leaf extract also led to an increase in cancer cell death (approximately 40–50%). Cancer cells under oxidative stress co-treated with all corn leaf extracts (except the chloroform extract) showed cytotoxicity similar to that of the hydrogen peroxide-treated groups (approximately 50% and 70%). This indicated that the aqueous and methanolic leaf extracts did not influence the cytotoxic action of hydrogen peroxide on cancer cells. Therefore, various apoptosis-related events in Hep2 cells exposed to the leaf extract showed potential anticancer activity of corn leaves.

Subsequently, Sasaki et al. [48] prepared and evaluated the effect of corn-derived nanoparticles against colon tumor cells (murine colorectal carcinoma) and macrophage-like immune cells (RAW264.7) after 24 h of incubation. It was observed that maize-derived nanoparticles were absorbed by colon26 cells and RAW264.7 cells, with a selective reduction in colon26 cell proliferation. Furthermore, maize-derived nanoparticles induced the release of tumor necrosis factor-α from RAW264.7 cells. The combination of maize-derived nanoparticles and RAW264.7 significantly suppressed colon26/fluc cell proliferation. Daily intratumoral injections of maize-derived nanoparticles significantly suppressed the growth of subcutaneous colon26 tumors in mice, without significant body weight loss. These results indicate the antitumor activity of maize-derived nanoparticles.

In 2023, Sasaki et al. [49] aimed to increase the applicability of intratumoral injection of maize-derived nanoparticles in cancer treatment by examining the tissue distribution of maize-derived nanoparticles after intravenous injection in mice. They modified the surface of the nanoparticles with polyethylene glycol (PEG) to improve tumor delivery and examined the tissue distribution and anticancer activity of PEG-maize-derived nanoparticles in mice with tumors. They determined that a PEG:maize-derived nanoparticle ratio of 50 was optimal for the preparation of PEG-maize-derived nanoparticles. Repeated intravenous injections of PEG-maize-derived nanoparticles significantly slow tumor growth, without significant hepatotoxicity or nephrotoxicity, indicating that controlling the tissue distribution of maize-derived nanoparticles through PEG surface modification could be a promising strategy for developing intravenously injectable maize-derived nanoparticles for cancer therapy. In addition to these studies, evaluations have also been conducted on other cell lines, such as HeLa (cervical cancer) (Table 2).

Both the blue corn extract and the native tortilla extract showed cytotoxic activity, achieving inhibitions of 61.04 and 68.69% at 1000 μg/mL, respectively. Although the activity was moderate compared to other vegetables, the results suggest that traditional tortilla processing does not eliminate the bioactive compounds. Phytochemical analysis by HPLC-ESI-MS identified 28 cyanidin-derived compounds, including cyanidin-3-glucoside, acylated anthocyanins, and oligomeric proanthocyanidins. Antioxidant activity, determined by DPPH and TBARS, showed a significant capacity to inhibit lipid peroxidation, suggesting that the anticancer potential may be partially related to the reduction of cellular oxidative damage. However, no studies of selectivity or specific mechanisms of apoptosis or cell arrest were reported [50].

### 3.3. Wheat

*Triticum* ssp. is the scientific name for wheat, a large crop and one of the most important food grains in the cereal family [22,59]. Wheat production exceeded 800 million tons in 2022, with China, India, and Russia being the main producers, according to FAO and USDA data [60,61]. In traditional medicine, wheat and its juice were used to strengthen the immune system and treat various ailments [22,52]. A few studies have begun to address its effect on various cancers, as shown in Table 2. The information presented for wheat indicates that its extracts show moderate anticancer potency compared to other cereals, such as barley. Notably, the ethanolic extract not only induces cell growth inhibition in HepG2 cell line (IC_50_ = 138.02 µg/mL), but also promotes specific mechanisms of action, such as apoptosis. Furthermore, it exhibited significant antioxidant activity using DPPH, FRAP, and superoxide radical scavenging assays. HPLC analysis identified compounds such as coumarin, cinnamic acid, and indole derivatives [53]. The aqueous extract showed activity against Caco-2 and HepG2 (IC_50_ = 247.6 and 226.4 µg/mL, respectively), suggesting lower biological activity. Furthermore, modulation of key proteins in cell cycle regulation and tumor survival is observed, such as increased p53 and decreased BCL2, cyclin D1, MMPs, and VEGF, suggesting a more complex and targeted antitumor effect [53]. The ethanolic extract of grass showed activity against oral cancer KB cells, with an IC_50_ of 156 μg/mL [51], while the methanolic extract exhibited nearly 50% inhibition against SKBR3 and SKOV3 at 600 μg/mL. Although potency was moderate, phytochemical studies revealed the presence of phenols, flavonoids, tannins, alkaloids, coumarins, sterols, and terpenoids, metabolites widely associated with antioxidant and antiproliferative properties [52]. Although detailed selectivity studies were not reported, the observed mechanisms indicate that wheat possesses anticancer potential associated with apoptotic regulation and antioxidant activity.

### 3.4. Barley

Barley (*Hordeum vulgare* L.) is one of the most widely cultivated cereals globally, with an annual production exceeding 150 million tons, the European Union being the main producing region [62]. Barley is widely used as a dietary supplement, a raw material for baking and brewing, animal feed, and as a medicinal ingredient in traditional medicine [63,64,65]. Studies have shown that barley-containing products have anticancer effects [66,67]. The effect of barley water and juice extracts on cancer cell lines has also been demonstrated (Table 2). Aqueous extracts show low IC_50_ values (0.7–1.3 µg/mL) in cell lines such as HT-29 and A549, indicating high cytotoxic efficacy even at very low concentrations. Similarly, juice-type extracts also exhibit biological activity, although slightly lower, with IC_50_ values between 2.4 and 7.4 µg/mL. The mechanism of action involved cell necrosis identified by confocal microscopy with PI staining. Although necrosis can represent a potent form of cell death, it may also be associated with lower biological specificity compared to programmed apoptosis. The extract also showed antioxidant capacity as determined by DPPH. However, no specific metabolites responsible for the activity were identified, nor were molecular mechanisms more detailed described [54].

Recently, Abuarab and Talib [68] evaluated the antitumor and antiproliferative activity of ethanol, *n*-hexane, aqueous/methanol, and water extracts obtained from barley bran against the MCF-7, A549, and HCT-116 cell lines (human colorectal cancer). The barley bran extracts inhibited cancer cell proliferation in a dose-dependent manner at a concentration of 5 mg/mL. According to immunoassays, the *n*-hexane and aqueous/methanol extracts significantly increased lymphocyte proliferation and macrophage pinocytosis activity. Phagocytosis activity was increased with the *n*-hexane and ethanol extracts. Furthermore, in vivo studies showed that the average tumor size and weight of mice receiving the 30% barley bran group were significantly reduced compared to the control group. During the study, higher levels of Th1 cytokines (IFN-γ, IL-2) and lower levels of Th2 cytokines (IL-4) and T cell regulatory cytokines (IL-10) were observed due to the consumption of barley bran in the diet. This demonstrates how barley bran can be used as a prophylactic agent due to its potential anticancer and immunomodulatory activity.

### 3.5. Rye

Rye (*Secale cereale*) has the highest dietary fiber content among cereals [69]. The inclusion of rye in a balanced, nutrient-rich diet has been described as potentially contributing to the prevention of various chronic diseases [70,71]. In cancer, whole rye has been shown to have favorable effects on prostate cancer progression in animal models, while in humans, consumption of rye bread with bran has been shown to increase apoptosis [72,73,74].

In 2010, Landberg et al. [75] investigated the effect of high intake of whole rye and bran on prostate cancer progression, assessed by prostate-specific antigen (PSA) levels, in men diagnosed with prostate cancer. Their results showed that total plasma concentrations of prostate-specific antigen were lower after treatment with wholegrain rye and bran compared to refined wheat with added cellulose, with a mean treatment effect of −14%. Furthermore, fasting insulin levels and 24 h urinary C-peptide excretion were lower after treatment with wholegrain rye and bran compared to refined wheat.

After Zamaratskaia et al. [76] evaluated the effects of wholegrain rye and bran consumption on low-grade inflammation and biomarkers of endothelial function in men with prostate cancer. They observed that tumor nucleolus receptor 2, E-selectin, and endostatin concentrations were significantly lower after consumption of the rye diet compared to the control diet (refined wheat). Cathepsin S concentration was positively correlated with tumor nucleolus receptor 2 and endostatin concentrations in all cases. Strong and consistent correlations were observed between intercellular adhesion molecule-1 and vascular cell adhesion molecule-1, and between interleukin-6 and the interleukin-1 receptor antagonist. These effects were accompanied by a reduction in prostate-specific antigen.

### 3.6. Sorghum

Sorghum (*Sorghum bicolor*) is the fifth most-consumed cereal in the global human diet, with a high energy value [77,78,79,80]. Global production of this cereal is approximately 60 million tons annually, with the United States, Nigeria, Sudan, Mexico, and India being the largest producers [81]. Furthermore, sorghum consumption has been described as potentially beneficial for cancer prevention [82,83].

In 2012, Park et al. [84] evaluated the anticancer activity of *Hwanggeumchal sorghum* extract against the MDA-MB 231 and MCF-7 cell lines. Their results predicted that specifically blocking STAT5b in breast cancer cells using sorghum extract could enhance apoptosis of solid cancer cells. Furthermore, in vitro and in vivo treatment of breast cancer cells with sorghum extract induced growth arrest and apoptosis, along with blocking constitutively active JAK/STAT signaling pathways. They also observed that sorghum extract can block angiogenesis by modulating the STAT3/VEGF pathway and VEGF-R2 levels in MDA-MB 231 and MCF-7 cancer cells. Finally, sorghum extract also inhibited metastasis in MDA-MB 231 and MCF-7-induced metastatic animal models. In 2019, Rao et al. [57] also described antiproliferative activity of phenolic sorghum extracts (Table 2). The phenolic extract of the Shawaya short black 1 variety (with black pericarp) showed approximately 30% inhibition in the SW480 cell line, suggesting a relevant cytotoxic effect, although less potent than other reported extracts. However, its importance lies in its mechanism of action, as it induces cell apoptosis by activating caspases 3 and 7, as well as increasing the expression of the p53 protein, a key regulator of the cell cycle and programmed cell death (determined by flow cytometry).

### 3.7. Oats

Oats (*Avena sativa* L.) are another of the world’s most important cereals due to their high nutrient content and global production of around 22 million tons [57,85,86]. This cereal has been used in the treatment of various conditions and diseases, such as inflammatory and cardiovascular diseases [87,88,89]. Recently, research has begun on the antiproliferative potential of oats, encouraging further studies in cancer (Table 2). Table 2 shows that the ethanolic extract of oat seed exhibits moderate activity in Caco-2 cells, with an IC_50_ of 67.0 μg/mL at 24 h, decreasing at 48 h (IC_50_ = 11.0 μg/mL). The mechanism involved apoptosis, determined by DAPI staining. When the ethanolic extract of oats was combined with 5-fluorouracil, the concentration that eliminated 50% of the cells for each compound separately was ~122 and 249 μg/mL, respectively. The combined dose was reduced to ~86 μg/mL. The combination index (CI < 1) was synergistic in all combinations evaluated [56]. Additionally, the aqueous extract shows high inhibition (94.3%) in the MCF-7 cell line, suggesting marked efficacy in this specific model. HPLC analysis identified benzoquinones, anthraquinones, salicylic acid, cinnamic acid, flavonoids, and tannins [57].

### 3.8. Sugarcane

Sugarcane (*Saccharum* spp.) is a perennial grass widely cultivated throughout the world [90,91,92]. It is also of great economic importance for food production and processing [93]. Sugarcane accounts for approximately 80% of global sugar production, with Brazil, India, China, and Thailand being the largest producers worldwide [94]. Among its byproducts, the leaves are valued for their medicinal properties and have long been consumed as tea [58,95,96]. Furthermore, sugarcane leaf extract has been described as exhibiting antioxidants, antibacterial, and antitumor activities [95,97,98].

Studies have also been conducted using sugarcane extract (Table 2), which showed consistent anticancer activity in various cell lines with GI_50_ values ranging from 5.9 to 52.4 µg/mL. Its efficacy in multidrug-resistant cells (NCI-ADR/RES) stands out, with a GI_50_ of 5.9 µg/mL, suggesting potential against tumor resistance mechanisms. However, selectivity was limited, as normal keratinocytes (HaCat) exhibited a GI_50_ of 26.1 µg/mL, similar to several tumor cell lines. GC-MS (Gas chromatography-mass spectrometry) analysis identified β-carotene, sitosterol, stigmasterol, campesterol, α-tocopherol, ferulic acid, tricin, and other phenolic compounds and phytosterols. These metabolites possess known antioxidant, anti-inflammatory, and antiproliferative properties [50].

Although currently there is limited information available on the antiproliferative activity of sugarcane extract, it is a cereal of great nutritional importance that warrants further investigation.

### 3.9. Overview

Studies conducted on species of the Poaceae family demonstrate that solvent polarity significantly influences the recovery of bioactive metabolites and, consequently, the observed anticancer activity. In general, ethanolic and methanolic extracts showed greater cytotoxic activity and a better capacity to induce apoptosis compared to aqueous extracts, likely due to their ability to extract phenolic compounds, flavonoids, anthocyanins, and lipophilic metabolites with antiproliferative properties. In rice, for example, ethanolic extracts of bran exhibited pro-apoptotic, anti-angiogenic, and immunomodulatory effects associated with the presence of ferulic acid, *p*-coumaric acid, saponarin, and FAHFAs, while aqueous extracts showed lower cytotoxicity despite having a higher total phenolic content. This suggests that bioactivity depends not only on the number of phenolic compounds extracted, but also on the chemical nature and bioavailability of specific metabolites. Furthermore, less polar fractions, such as hexane and dichloromethane, showed significant anti-invasive activity related to the inhibition of metalloproteinases and collagenases, demonstrating that lipophilic compounds also play a relevant role in antimetastatic activity [33,36]. A similar trend was observed in wheat and oats [51,53,56,57]. These differences confirm that solvent selection influences the recovered phytochemical profile and can substantially modify the biological response.

Among the most promising species in this family, barley stood out, with its aqueous extracts and juices exhibiting the lowest reported IC_50_ values, reaching concentrations of 0.7–7.4 µg/mL in HT-29 and A549 lines. Although the described mechanism was primarily associated with cell necrosis, these results suggest high biological potency. Furthermore, in vivo studies demonstrated that dietary consumption of barley bran significantly reduced tumor size and promoted a favorable immunomodulatory response, characterized by an increase in Th1 cytokines and a reduction in Th2 and regulatory cytokines [54]. On the other hand, sugarcane showed consistent activity against multiple tumor cell lines, including multidrug-resistant cells, with particularly low GI_50_ values in NCI-ADR/RES cells, suggesting significant potential against tumor resistance mechanisms [58]. Rice also showed outstanding results due to the diversity of mechanisms involved and the wide range of extracts evaluated. Phenolic extracts of leaves and bran induced apoptosis via mitochondrial pathways and death receptors, accompanied by the inhibition of PI3K/Akt and Erk, two essential pathways for tumor proliferation and survival [27,31,32,33,34,35,36].

The mechanisms of action reported in Poaceae species primarily included apoptosis, cell cycle arrest, angiogenic inhibition, regulated oxidative stress, and immunomodulation. Caspase activation, increased p53 expression, decreased BCL2 and VEGF expression, and inhibition of metalloproteinases and the PI3K/Akt pathway were recurring events in several species. In sorghum, for example, phenolic extracts activated caspases 3 and 7 and promoted p53 expression, in addition to modulating the STAT3/VEGF pathway, which is associated with angiogenesis and metastasis. In oats, the combination of the ethanolic extract with 5-fluorouracil showed significant synergistic effects, highlighting the potential of these cereals as adjuncts to conventional therapies [55,56,57].

Finally, the cereals studied exhibit significant nutraceutical potential due to their richness in flavonoids, anthocyanins, phytosterols, phenolic acids, and tocopherols. Metabolites such as ferulic acid, tricin, γ-oryzanol, γ-tocotrienol, cyanidin-3-glucoside, quercetin, and luteolin possess antioxidant, anti-inflammatory, and chemopreventive properties. Regular consumption of whole grains, bran, and minimally processed products could contribute to reducing oxidative stress, modulating inflammatory processes, and decreasing factors associated with carcinogenesis. In particular, the consumption of brown rice, barley, oats, and rye within dietary patterns rich in fiber and antioxidants could represent a complementary nutritional strategy for the prevention of chronic diseases and cancer. Furthermore, the fact that several agricultural by-products, such as bran, leaves, and husks, exhibit relevant biological activity highlights their potential for the development of functional foods, nutraceuticals, and bioactive compounds with future therapeutic applications.

## 4. Solanaceae Family

All the consumed species in this family are native to the Americas. They generally have a high yield per hectare (approximately 28 million) and include many globally important food crops, such as peppers, tomatoes, eggplants, and potatoes; and they play an important role in nutrition and health [99,100,101].

### 4.1. Tomato

The tomato plant is seasonal, and its fruit (*Solanum lycopersicum* L. Moench) can be eaten fresh, juiced, or used as an ingredient in savory dishes. It is also processed to obtain tomato paste [102,103]. It is one of the most consumed foods in the world, with Turkey, the United States, and Mexico being the largest producers [104]. Furthermore, its important medicinal properties as an antioxidant, anti-inflammatory, and anticancer agent have been demonstrated [102,105,106,107].

Table 3 presents recent research on extracts or nanoparticles based on this fruit and their effects on cancer cell lines. These studies highlight the methanolic extract, derived from the root, which exhibits high cytotoxicity with IC_50_, even at concentrations < 15 μg/mL, in some cases, against MCF-7, Caco-2 and HepG2 cell lines. In silico analyses indicated the involvement of apoptotic pathways, p53 signaling, and TNF. Furthermore, molecular docking studies suggested that inosine and chlorogenic acid could act as inhibitors of HDAC2, an important target in epigenetic cancer therapies. The metabolomic profile identified flavonoids, terpenoids, and phenolic compounds such as quercetin, resveratrol, rutin, dihydromyricetin, syringic acid, and δ-tocopherol (by HPLC-HESI-HRMS). Root-derived nanocrystals partially increased cytotoxic activity against Caco-2 and HepG2, further confirming the potential of nanoformulations [108]. The methanolic extract also showed significant activity against the HeLa, H2228, and H3122 cancer cell lines. Less inhibition (~45%) was observed in normal HEK293 cells, suggesting moderate selectivity. This activity was associated with apoptosis, as determined by DAPI staining and an Annexin V, and propidium iodide (PI) assay in HeLa cells [109].

The ethanolic extract showed significant activity against HepG2 (IC_50_ = 48 μg/mL) with favorable selectivity, since the IC_50_ in normal 3T3 fibroblasts was greater than 200 μg/mL. The mechanism involved apoptosis, DNA damage, and cytochrome c release, indicating activation of the intrinsic mitochondrial pathway (confirmed by AO/PI double staining and DNA fragmentation analysis). HPLC-DAD-MS analysis primarily identified caffeic acid, gallic acid, chlorogenic acid, vanillic acid, quercetin, kaempferol, naringin, and epigallocatechin gallate. These metabolites possess widely reported antioxidants and pro-apoptotic properties [112]. The chloroform extract showed moderate activity against AGS and HCT-116 cells, with IC_50_ values of 55.18 and 70.73 μg/mL, respectively. The mechanism of action involved apoptosis and cell cycle arrest in the G2/M phase, determined by flow cytometry and the Annexin V-FITC assay. Metabolomic analysis revealed various phenolic compounds and carotenoids identified by UHPLC-HRMS and UV-Vis spectroscopy, including glycosylated phenolic derivatives, amino acids, and organic compounds such as citric acid and leucinopine, which may act synergistically in the antiproliferative activity [110].

Zinc oxide nanoparticles synthesized from tomato juice showed approximately 50% inhibition of HepG2, while in lung fibroblasts (WI-38) the inhibition was approximately 25%, suggesting some tumor selectivity. Furthermore, the nanoparticles exhibited moderate antioxidant activity in DPPH assays [111].

Taken together, the results indicate that tomato possesses multifactorial anticancer activity associated with phenolic compounds, flavonoids, and carotenoids. The predominant mechanisms include apoptosis, cell cycle arrest, and regulation of epigenetic and cell signaling pathways.

### 4.2. Eggplant

Eggplant (*Solanum melongena* L.) is a vegetable used in the contemporary cuisines of many countries and is consumed cooked or processed [101,123]. Eggplant ranks fifth in economic importance among solanaceous crops, with China being the largest producer at 35.5 million tons per year [124,125].

Furthermore, some studies have been conducted using extracts of this vegetable to evaluate their anticancer potential (Table 3). The anticancer activity reported in Table 3 depended on the plant part and the type of extract used. The methanolic extract of the peel showed the greatest potency, against HepG2, HCT116, HEP2, MCF7, and HELA, with IC_50_ values between 2.14 and 6.56 μg/mL. Phytochemical analyses allowed the isolation of steroidal alkaloids such as solasodine, solamargine, and solasonine, as well as steroidal glycosides such as β-sitosterol-3-*O*-β-_D_-glucoside and poriferasterol glucoside (determined by ^1^H-NMR, ^13^C-NMR, COSY, and HMBC). These metabolites are widely recognized for inducing apoptosis, altering cell membranes, and modulating tumor proliferation. Furthermore, this extract showed in vivo activity against CCl4-induced hepatocellular carcinoma in rats, reducing α-fetoprotein and restoring hepatic markers such as AST (aspartate aminotransferase), ALT (alanine aminotransferase), and albumin, supported by histopathological analysis. This suggests significant hepatoprotective and anticancer potential [113].

Meanwhile, the methanolic extracts of leaves and branches showed significant activity against AGS and Caco-2 with GI_50_ values close to 12 and 17 μg/mL. It also exhibited anti-inflammatory activity by decreasing IL-6 and inhibiting nitric oxide. HPLC-DAD-MS analysis identified numerous caffeoylquinic acid derivatives, glycosylated flavonoids of quercetin, kaempferol, and isorhamnetin. These metabolites possess recognized antioxidant, anti-inflammatory, and pro-apoptotic activities [101]. Ethanolic extracts of the seeds exhibited activity against various cancer cell lines, including colon, glioblastoma, neuroblastoma, and pancreatic cancer, with IC_50_ values between 23 and 49 μg/mL. The mechanism involved apoptosis mediated by PARP1 degradation, autophagy, and disruption of cellular microtubules, accompanied by an increase in LC3β-II. Furthermore, the extract reduced colonic polyps in in vivo models of colon cancer stem cells. Identified metabolites included salannin, kaempferol sophorotrioside, and capsianoside II (determined by QTOF-QTOF) [115]. Finally, silver nanoparticles synthesized from fresh leaves showed moderate inhibition of MDA-MB-231 (46.77%). The mechanism involved delayed apoptosis, determined by Annexin-V/FITC-PI. HPTLC analysis revealed phenolic compounds, flavonoids, anthocyanins, saponins, and alkaloids, which likely contribute to the observed activity [114].

### 4.3. Pepper

The pepper (*Capsicum annuum* L.) is a widely consumed species worldwide, with a production of 42.282 million tons in 2020, with China being the main producer [126]. Furthermore, the pepper is recognized for its pharmacological properties [127,128]. Among these is its antiproliferative activity against cancer cell lines (Table 3). Ethanolic extracts of sweet and hot peppers and hydroethanolic extracts of peppers showed moderate activity against PC-3, MDA-MB-231, and LoVo cells, respectively [116,119]. Ethanolic extracts of sweet pepper and chili pepper showed IC_50_ values of 78 and 68 μg/mL against PC-3, respectively. In murine L929 fibroblasts, the IC_50_ values were close to 90 μg/mL, indicating limited selectivity. LC-QTOF-MS analysis identified compounds such as gallic acid, capsianoside VII, capsidiol, capsiate, and luteolin [116]. The dry hydroethanolic extract, in addition to exhibiting moderate activity against MDA-MB-231 and LoVo (IC_50_ < 200 µg/mL), showed antioxidant potential determined by DPPH, ABTS, and FRAP. UHPLC-HRMS/MS analysis identified 79 phenolic metabolites, including quercetin, rutin, apigenin, biochanin A, tricin, capsaicin, dihydrocapsaicin, capsiate, and various phenolic acids. These metabolites possess widely reported antioxidant, anti-inflammatory, and antiproliferative properties [119].

While the crude extract exhibited relatively weak cytotoxicity against several tumor cell lines (MCF-7, MDA-MB-231, K562, PANC1, and A375), with IC_50_ values between 153 and 393 µM, normal fibroblasts showed lower sensitivity (IC_50_~721 µM), suggesting some selectivity. HPLC analysis confirmed the presence of capsaicin as the main bioactive metabolite [117]. In contrast, the aqueous leaf extract showed a better response against A549 cells (IC_50_ = 85.45 μg/mL). Furthermore, flavonoids, alkaloids, phenols, carbohydrates, and tannins were identified in this extract. Interestingly, the incorporation of this extract into metallic nanoparticles progressively improved its cytotoxic activity, especially with Ag-Au nanoparticles (IC_50_ = 57.35 μg/mL). This activity was associated with the induction of apoptosis and cell necrosis, as determined by AO/EB and flow cytometry [118]. This suggests that nanoformulation could enhance bioavailability or cell interaction (Table 3).

### 4.4. Potato

The potato (*Solanum tuberosum* L.) ranks third in agricultural importance for human consumption, after rice and wheat [129,130]. In 2021, 376 million metric tons of potatoes were produced globally, with China being the leading producer [131]. Because potatoes are widely consumed worldwide, their potential health benefits could have broad applications, including inhibitory properties against cancer cells [132]. Table 3 presents recent research on potato-based extracts and nanoparticles with biological activity against cancer cell lines. In *Solanum tuberosum* L., the observed anticancer activity was highly dependent on the preparation method. While the methanolic leaf extract showed limited cytotoxic activity (IC_50_ between 139 and 351 μg/mL) against breast cancer cell lines, it exhibited favorable selectivity, as the IC_50_ in normal HUVEC cells was 689.9 μg/mL. Phytochemical analysis revealed elevated levels of phenolic acids and flavanols, including chlorogenic acid, caffeic acid, protocatechuic acid, and rutin, as well as glycoalkaloid precursors [120].

Nanostructured systems derived from the peel exhibited considerably more promising behavior. Silver nanoparticles obtained from the peel extract showed relevant cytotoxicity (IC_50_ 4.31–23.37 μg/mL) against glioma, colon, and ovarian cells. Furthermore, they exhibited favorable selectivity against human dermal fibroblasts (IC_50_ = 43.39 μg/mL). This suggests that nanoformulation significantly improves the biological efficacy of the potato [121]. In contrast, selenium nanoparticles showed much lower activity, as high concentrations (2000 μg/mL) were required to achieve 50% inhibition of A549 and OVCAR-3 [122]. Taken together, these results indicate that, although the potato exhibits modest activity, its by-products, particularly the peel, could acquire greater pharmacological value through nanobiotechnology approaches (Table 3).

### 4.5. Overview

In general, studies conducted on species of the Solanaceae family demonstrate that methanolic, ethanolic, and hydroethanolic extracts showed greater cytotoxicity than aqueous extracts, due to their ability to extract flavonoids, phenolic acids, steroidal alkaloids, and carotenoids with recognized antiproliferative properties [101,108,112,113,115]. For example, methanolic extracts of tomato and eggplant exhibited some of the lowest reported IC_50_ values, while aqueous nanoparticles generally showed lower potency, although in some cases they retained antioxidant activity and tumor selectivity [108,113]. Furthermore, the use of nanoparticles derived from plant extracts considerably increased cytotoxic activity [113].

Among the most promising species, eggplant stood out, particularly the methanolic extract of its peel, which exhibited high potency against multiple tumor cell lines with IC_50_ values between 2.14 and 6.56 μg/mL. This activity was associated with steroidal alkaloids such as solasonine, solamargine, and solasodine, metabolites known to induce apoptosis and alter tumor proliferation processes [113]. Eggplant also showed consistent anticancer activity associated with phenolic compounds and flavonoids such as quercetin, chlorogenic acid, rutin, and resveratrol [98,99,100,101,102,103,104,105,106,107,108,109,110,111,112,113,114,115]. Tomato extracts induced apoptosis, DNA damage, and cell cycle arrest, primarily in the G2/M phase, in addition to modulating pathways related to p53 and TNF [114,115]. On the other hand, potato peel-derived nanoparticles showed a notable improvement in cytotoxic activity compared to conventional extracts, achieving low IC_50_ values and favorable selectivity against normal cells [121].

The mechanisms of action reported in species of this family mainly included apoptosis, cell cycle arrest, autophagy, inflammatory modulation, and mitochondrial damage. Caspase activation, PARP1 degradation, cytochrome c release, and regulation of proteins related to tumor proliferation were frequent events in several studies [109,110,111,114,123,126]. Furthermore, some extracts showed anti-inflammatory effects by decreasing IL-6 and nitric oxide, which could contribute to limiting tumor progression [101]. In chili peppers, the presence of capsaicin, luteolin, and other phenols was associated with antioxidant and antiproliferative properties, although with moderate activity compared to tomato and eggplant [116,117,118,119].

Finally, the results support the nutraceutical potential of the Solanaceae family species due to their richness in carotenoids, flavonoids, phenolic compounds, and bioactive alkaloids. Metabolites such as lycopene, quercetin, chlorogenic acid, capsaicin, rutin, and δ-tocopherol possess antioxidant, anti-inflammatory, and chemopreventive properties. Therefore, regular consumption of tomatoes, eggplants, chili peppers, and potatoes within balanced diets could contribute to reducing oxidative stress and modulating processes associated with carcinogenesis. Furthermore, the utilization of byproducts such as peels and seeds represents a promising strategy for the development of functional and nutraceutical foods with potential applications in cancer prevention.

## 5. Fabaceae Family

Legumes, or Fabaceae, are the second most economically important family in terms of human nutrition, after Poaceae [133,134]. Legume seeds are characterized by their nutritional value and compared to other plants, have a high protein content [135]. Inclusion in the diet and regular consumption of legumes are associated with numerous health benefits [136,137,138,139].

### 5.1. Pea

The pea (*Pisum sativum* L.) is a leguminous plant cultivated worldwide, ranking third in production among pulses. Canada is the largest producer, followed by China, Russia, and India, making it a staple crop in many regions globally [140,141,142].

Pea seeds, commonly known as green peas, are valued as a low-cost food source. Their various pharmacological properties have also been investigated [106,143,144,145,146]. Some studies have also been conducted on extracts from the pea pods, stems, and leaves (Table 4).

The ethyl acetate extract of the peel showed 73.6% inhibition of MCF-7 cells and an LC_50_ of 4.31 μg/mL, indicating cytotoxic potency. The mechanism of action involved apoptosis mediated by increased Bax and decreased Bcl-2, determined by ELISA, suggesting activation of the mitochondrial apoptotic pathway. Furthermore, the extract exhibited moderate antioxidant capacity as measured by DPPH. HPLC-DAD analysis identified several metabolites, including flavonoids, hesperidin, pyrogallol, catechol, and *p*-hydroxybenzoic acid [147]. Sulfated extracts derived from the peel showed cytotoxic activity against HepG2 and MCF-7. The sulfated soluble alkaline extract showed an IC_50_ of 26.9 μg/mL against HepG2, while the acid-insoluble, neutral-soluble, and alkaline-soluble fractions showed IC_50_ values close to 10 μg/mL against MCF-7. These extracts also exhibited high antioxidant capacity, achieving free radical neutralization between 91–98% for non-sulfated extracts. Chemical analysis identified sulfated carbohydrates and monosaccharides such as uronic acid, galactose, glucose, arabinose, and xylose, suggesting that sulfated polysaccharides could contribute to the observed biological activity [148].

The ethanolic extract of leaves and stems showed significant activity against MCF-7 (IC_50_ = 17.67 μg/mL), although lower than against doxorubicin (IC_50_ = 2.69 μg/mL). The metabolomic study using LC-ESI-QTOF-MS/MS identified 98 metabolites, mainly flavonoids and phenolic derivatives such as kaempferol-*O*-hexuronide, galangin, tricin, liquiritigenin, daidzein, *p*-coumaric acid, ferulic acid, and caffeic acid [149]. The ethanolic extract of pods showed more moderate activity against MCF-7 (IC_50_ = 32.92 μg/mL). However, the overall results suggest that pea has significant anticancer potential, mainly related to flavonoids and bioactive polysaccharides capable of inducing apoptosis and modulating oxidative stress.

### 5.2. Bean

The common bean (*Phaseolus vulgaris* L.) is one of the most widely consumed legumes worldwide, with a production of 27.5 million metric tons in 2020. Asia is the largest producer, and it is characterized by its high nutritional value and is a fundamental component of the human diet [158,159,160]. Furthermore, several studies have indicated that bean consumption can prevent and/or control various chronic degenerative diseases [161,162,163], including cancer [164,165].

In this regard, Campos-Vega et al. [166] obtained total dietary fiber extracts from beans, which were fermented with human gut flora to simulate the digestion of total dietary fiber extracts in the large intestine. Subsequently, they exposed HT-29 cells to different concentrations of the 100% fermented extract. The results showed that the extract fermented with human gut flora inhibited HT-29 cell growth by 64%. Furthermore, they evaluated the expression of genes related to p53-mediated signal transduction. Of these, the apoptosis genes SIAH1, PRKCA, and the downregulation of the cell cycle gene MSH2 were the most overexpressed (30.5, 18.4, and 9.8 times, respectively), while the cell cycle genes CHEK1 and GADD45A were downregulated (21.4 and 9.1 times, respectively). These results provided information on the mechanism underlying the chemoprotective function of total dietary fiber extract from beans against colon cancer.

Subsequently, Bernardi et al. [167] analyzed two varieties of cooked beans (Cannellino and Piattellino). These were subjected to simulated in vitro gastrointestinal digestion, and their potential as anticancer agents was evaluated using an MTT assay on HT29 and HCT116 cell lines. The analyses demonstrated that the extract obtained after gastrointestinal digestion of cooked beans induces cell death through the induction of autophagy. The authors demonstrated that an extract obtained after gastrointestinal digestion of cooked Cannellino and Piattellino beans at a concentration of 100 μg/mL reduces cell viability in both HT29 (88.41 and 94.38%, respectively) and HCT116 (86.29 and 91.23%, respectively) cell lines. Furthermore, the digested cooked bean extracts did not affect the viability of human fibroblasts or keratinocytes. Chloroform extract was also recently analyzed, which showed high inhibition of cell growth and a CC_50_ of 3.84 μg/mL in the MCF-7 cell line, even better than doxorubicin (CC_50_ = 30.03 μg/mL), indicating high activity compared to other plant extracts. GC-MS analysis identified several phytochemicals, with 8-pentadecanol, lupeol, and 2-butoxyethyl oleate standing out as the main metabolites. Lupeol has been widely reported for its antiproliferative and proapoptotic properties in various tumor models, including regulation of apoptosis and suppression of inflammation [150]. However, further analysis is needed to clarify its mechanisms of action on tumor cells (Table 4).

### 5.3. Chickpea

The chickpea (*Cicer arietinum* L.) is a staple food in the human diet, ranking third among the most important legumes worldwide with an annual production of 12.8 million tons, with India being the leading producer [168,169]. Furthermore, chickpeas have been described as having a high content of nutrients with potential health benefits, especially in metabolic and chronic diseases such as cancer [170,171,172].

In 2023, Cid-Gallegos et al. [173] evaluated the chemopreventive effect of chickpeas on the progression of colon carcinogenesis induced by azoxymethane and dextran sodium sulfate in a murine model at 1, 7, and 14 weeks post-induction. In addition to evaluating the expression of biomarkers in the colon of BALB/c mice fed diets supplemented with 10% and 20% cooked chickpeas, the results showed that a diet with 20% cooked chickpeas significantly reduced tumors and biomarkers of proliferation and inflammation in mice with colon cancer. They also observed that body weight loss decreased (increasing by 47% and 35% with both diets) compared to the positive control group (a 29% decrease in body weight). Finally, the tumor reduction was most evident in week 7 in the groups fed a diet with 20% cooked chickpeas (46%), demonstrating that diets with 10% and 20% cooked chickpeas exert a chemopreventive effect. Furthermore, chickpea-based extracts and nanoparticles have been evaluated against breast cancer cell lines (Table 4). Germinated black chickpea extract showed relatively favorable cytotoxic activity (IC_50_ of 11.11 μg/mL) against MDA-MB-231 cells, suggesting that the germination process may enhance the antiproliferative effect. The mechanism of action was associated with apoptosis, determined by fluorescence microscopy using DAPI/phalloidin dual staining. Additionally, the extract exhibited significant antioxidant capacity. HPLC-IT-MS analysis primarily identified isoflavones and glycosylated derivatives, including isoformononetin glucoside, formononetin malonyl glucoside, biocanin-A glucoside, biocanin-A malonyl glucoside, formononetin, and biocanin-A. These metabolites are known to modulate apoptosis, oxidative stress, and hormonal pathways related to tumor proliferation [151].

In contrast, silver nanoparticles obtained from aqueous chickpea hull exhibited cytotoxic activity (IC_50_ = 82.01 μg/mL), albeit lower, against MCF-7 cells. LC-MS/MS analysis identified metabolites such as myricetin-hexosyl-deoxyhexoside, formononetin, and khellin [152]. Taken together, these results position chickpeas as a plant source of pharmacological interest, although further studies are needed to elucidate their mechanisms of action and compare the impact of processes such as germination and nanoformulation on their antitumor activity.

### 5.4. Faba Beans

Faba beans (*Vicia faba* L.) rank seventh in production with 7.79 million metric tons in 2022, with China being the largest producer, followed by Ethiopia and Australia [137,174]. In several countries, such as Egypt, both the pods and the beans are consumed as a main dish [155]. Extracts from both the pods and the beans have been described as having antioxidants, anti-inflammatory, and anticancer properties, among others [175,176,177].

In this regard, they have evaluated the antiproliferative potential of faba bean extracts on cancer cell lines, promoting the use of faba beans in the human diet for their health benefits (Table 4). *Vicia faba* extracts showed moderate to high cytotoxic activities depending on the genotype and type of extract used. Extracts from the Nura genotype showed greater activity against AGS and HepG2 cell lines (IC_50_ less than 0.2 mg/mL) compared to the Rossa genotype (IC_50_ of 1.04 and 1.59 mg/mL), demonstrating that genetic variability can affect the concentration and composition of bioactive metabolites. The extracts also showed high antioxidant capacity using DPPH, TEAC, ORAC, and CAA assays. HPLC analysis identified phenolic acids and traces of anthocyanins, metabolites widely associated with antioxidant and antiproliferative properties [153].

Furthermore, the ethanolic extract exhibited activity against melanoma (IC_50_ = 118.7 μg/mL) and was associated with interference in microtubule organization and induction of apoptosis, as confirmed by the TUNEL assay. Additionally, HPLC-DAD analysis identified vanillic acid, ellagic acid, chlorogenic acid, quercetin, *p*-coumaric acid, ferulic acid, and gallic acid [154]. Meanwhile, the methanolic extract of pods showed high inhibition (>95%) of PC3 and HepG2 cells, although at higher concentrations, and with relatively high IC50 (~125 μg/mL), indicating moderate activity [155].

### 5.5. Lentils

Lentils (*Lens culinaris* L.) are widely cultivated worldwide, covering 5.6 million hectares and producing 5.6 million tons in 2021. Canada is the largest producer, accounting for 44% of global production [178,179]. Lentils have been described as having a high content of protein, minerals, dietary fiber, and iron [180]. In traditional medicine, lentils are attributed with antidiabetic, antioxidant, hematopoietic, immunoprotective, cardiovascular, and anticancer properties [181,182]. Research supports the antiproliferative potential of lentil extracts and nanoparticles (Table 4). The lentil extract showed IC_50_ values of 16.1, 26.34, and 45.89 μg/mL compared to MCF-7, HCT-116, and HepG2, respectively. The mechanism of action involved apoptosis, determined by flow cytometry with Annexin V-FITC/PI dual staining. LC-ESI-TOF-MS analysis identified 22 secondary metabolites, including flavonoids, flavonoid glycosides, and anthocyanidin glycosides. Among the most relevant compounds were kaempferol-3-*O*-robinoside-7-*O*-rhamnoside, quercetin-3-_D_-xyloside, epigallocatechin, catechin gallate, datiscin, and esculin. Silver nanoparticles synthesized from the extract showed a significant improvement in cytotoxic activity, achieving IC_50_ values of 0.37, 0.35, and 0.1 μg/mL, respectively. These results demonstrate that the nanoparticles could significantly increase the bioavailability and efficacy of the active metabolites [160]. Although detailed selectivity studies have not been reported, the high potency observed suggests that lentils represent a promising source of bioactive anticancer compounds.

Furthermore, Di Turi et al. [183] recently prepared an acetonitrile–water extract and evaluated its cytoprotective effect against chemotherapeutic agents using mouse cells (osteoblasts, bone marrow cells, and muscle fibers) and HEK293 and SHSY5Y neuronal cell lines. The results showed that in osteoblasts treated with 2.5 mg/mL of doxorubicin, irinotecan, staurosporine, and dexamethasone, lentil extract increased cell survival to 112.43, 97.36, 114.45, and 154.04%, respectively. In bone marrow cells treated with doxorubicin, dexamethasone, and staurosporine, lentil extract increased cell survival to 145.40, 155.56, and 53.15%, respectively. In muscle fibers treated with doxorubicin and staurosporine, lentil extract did not show a cytoprotective effect, but rather a negative synergistic effect, increasing cytotoxicity. The authors suggest a possible use for potentiating cytotoxic action in muscle fiber tumors, assuming the same results obtained in healthy muscle fibers are obtained in tumorous muscle fibers. While HEK293 cells treated with doxorubicin, dexamethasone, cisplatin, and irinotecan showed a survival rate of 98.95 to 244%, no protective effect was observed in SHSY5Y cells when treated with cisplatin. On the other hand, a cytoprotective effect was shown when treated with irinotecan, doxorubicin, and dexamethasone, with 129, 57.92, and 95.99% survival at 24 h, respectively.

### 5.6. Soybeans

Soybeans (*Glycine max* L.) had a production of 382 million tons in 2021 [184]. The United States, Brazil, and Argentina are the main producers [185]. Soybeans have been incorporated into the human diet in many countries due to their nutritional content and protective function against human diseases [186,187]. Among these diseases is cancer [188,189]. Furthermore, research has been conducted in this area, such as that presented in Table 4 and the work carried out by Chang and Tan [190], where they evaluated the effects of four extraction methods (variations of soaking and grinding) combined with cooking of soy milks prepared from yellow soybeans (ProSoy variety with high protein content) on DU145 prostate cancer cells. The results showed that raw soy milk exhibited the best antiproliferative activity (IC_50_ of 4.9 to 8.5 mg/mL) against DU-145 cells, encouraging further research into the potential antiproliferative activity of soy.

### 5.7. Overview

In the Fabaceae family species analyzed, it was observed that solvent polarity could influence the recovery of bioactive compounds and the antiproliferative activity exhibited. In general, ethanolic, methanolic, and ethyl acetate extracts showed greater cytotoxic activity compared to aqueous extracts, probably due to their ability to extract flavonoids, phenolic acids, alkaloids, and lipophilic compounds associated with pro-apoptotic and antioxidant properties. For example, the ethyl acetate extract of pea pods showed high cytotoxicity against MCF-7 cells [147], while sulfated extracts showed significant antioxidant and antiproliferative activity related to bioactive polysaccharides [148]. Among the species in this family, the lentil stands out, whose extracts and derived nanoparticles showed outstanding activity, achieving low IC_50_ values against MCF-7, HCT-116, and HepG2 cells [156]. Similarly, the pea exhibited significant activity associated with flavonoids and sulfated polysaccharides capable of inducing apoptosis and modulating oxidative stress [147,148]. On the other hand, the common bean showed significant chemopreventive activity, particularly after simulated digestion and fermentation processes, suggesting that the gut microbiota can transform dietary compounds into metabolites with greater biological activity [166,167]. In chickpeas, the germination process increased the antiproliferative potential, possibly due to the increase in bioactive isoflavones such as formononetin and biochanin A [151].

The described mechanisms of action primarily included apoptosis, cell cycle arrest, autophagy, and regulation of pathways related to tumor proliferation and oxidative stress. Events such as increased Bax, decreased Bcl-2, activation of mitochondrial apoptotic pathways, PARP1 degradation, and alteration of cellular microtubules were observed. Furthermore, some extracts showed the capacity to modulate genes related to p53, inflammation, and cell survival [147,154,156,157]. In fermented soybeans, the inhibition of STAT3 and the regulation of JNK and Erk1/2 suggest more specific mechanisms associated with tumor signaling [157].

Finally, species of the Fabaceae family exhibit significant nutraceutical potential due to their richness in flavonoids, isoflavones, phenolic compounds, and bioactive polysaccharides. Metabolites such as quercetin, kaempferol, catechins, ferulic acid, formononetin, and biochanin A possess antioxidant, anti-inflammatory, and chemopreventive properties. In this regard, regular consumption of legumes such as lentils, peas, chickpeas, beans, and soybeans within balanced diets could contribute to reducing oxidative stress and modulating processes related to carcinogenesis. Furthermore, processes such as germination, fermentation, and nanoformulation could increase the bioavailability and biological efficacy of these compounds, although in vivo studies are still needed for validation. This could facilitate the development of functional foods and complementary dietary strategies for cancer prevention.

However, although the overall results obtained for Poaceae, Solanaceae, and Fabaceae species are promising, most of the evidence presented comes from in vitro cytotoxicity studies, which have limitations related to cell models and their inability to reproduce the complexity of the tumor microenvironment, systemic interactions, and pharmacokinetic processes that occur in living organisms. Furthermore, the effective concentrations reported in some studies may not be easily achievable through dietary intake or after administration of the extracts due to factors such as limited absorption, metabolism, tissue distribution, and low bioavailability of certain phytochemicals. Therefore, extrapolating these findings to in vivo models and clinical applications should be done with caution. In this context, future research involving animal models and well-designed clinical trials is needed to validate the efficacy and safety observed in vitro. Furthermore, a more thorough phytochemical characterization of the extracts is required, along with the identification of the compounds responsible for the biological activity and the implementation of standardization processes that will improve the reproducibility of the results and strengthen the translational potential of these plant resources.

## 6. Conclusions

The evidence compiled in this review demonstrates that various edible crops belonging to the Poaceae, Solanaceae, and Fabaceae families constitute a relevant source of secondary metabolites with potential anticancer activity. The analyzed studies show that extracts, fractions, and nanoparticles derived from these cereals, vegetables, and legumes can exert antiproliferative effects on different tumor cell lines through complex molecular mechanisms, including the induction of apoptosis, cell cycle arrest, modulation of oncogenic signaling pathways, and inhibition of processes associated with tumor progression, such as angiogenesis and metastasis. However, despite the growing number of experimental studies, most research is limited to in vitro models, with little validation in vivo models and clinical studies that would allow for a more precise establishment of therapeutic relevance. In this regard, the study of food crops as sources of anticancer nutraceuticals represents an opportunity for the development of new research focused on identifying the metabolites responsible for biological activity and characterizing their molecular targets, as well as on designing advanced formulations, including nanostructured systems that improve their stability and biological efficacy. Taken together, the integration of knowledge generated around these edible crops could significantly contribute to the development of innovative nutritional and pharmacological strategies aimed at cancer prevention and complementary treatment.

## Figures and Tables

**Table 1:** In vitro anticancer activity of rice extracts and fractions and their reported mechanisms of action.

Product Used	Cell Line	In Vitro Activity	Mechanism of Action and Other Parameters Evaluated	Reference
Rice (*Oryza sativa* L.)				
Callus suspension culture	RXF-393 (Kidney cancer)	Inhibition = 82% at 1 mg/mL at 96 h	Structural alteration and reduction of cancer cells	[31]
	SW620 (Colon cancer)	Inhibition = 71% at 1 mg/mL at 96 h		
Phenolic subfraction of the leaf	MCF-7 (Breast cancer)	IC_50_ = 95.33 μg/mL at 72 h	Induction of apoptosis in HepG2 cells through mitochondrial and death receptor pathways, accompanied by inhibition of the PI3K/Akt and Erk signaling pathways	[27]
	Caco-2 (Colorectal adenocarcinoma)	IC_50_ = 82.19 μg/mL at 72 h		
	HepG2 (Colorectal adenocarcinoma)	IC_50_ = 74.42 μg/mL at 72 h		
Bran extract	HepG2	IC_50_ = 144.6 μg/mL	ND	[32]
	SW620	IC_50_ = 116.9 μg/mL		
	KATO-III (Gastric carcinoma)	IC_50_ = 99.6 μg/mL		
	BT474 (Breast cancer)	IC_50_ = 94.4 μg/mL		
	ChaGo-K-1 (Bronchogenic carcinoma)	IC_50_ = 25.6 μg/mL		
Ethanolic bran extract	MCF-7	IC_50_ = 0.36 mg/mL	Ethanol and methanol extracts showed the greatest activity in inducing apoptosis and inhibiting angiogenesis, and they also reduced VEGF concentration (n-hexane extract was less active). Both extracts were also effective in enhancing immunity by activating lymphocyte and phagocyte proliferation and modulating cytokine levels, as demonstrated by a lymphocyte proliferation assay. Both extracts showed antioxidant activity (IC_50_ = 114 and 168 µg/mL), compared to ascorbic acid (IC_50_ 1.74 µg/mL)	[33]
	T47D (Breast cancer)	IC_50_ = 0.37 mg/mL		
	EMT6/P (Murine breast cancer)	IC_50_ = 0.71 mg/mL		
	MDA-MB-231	IC_50_ = 0.64 mg/mL		
*n*-Hexane extract of bran	MCF-7	IC_50_ = 0.12 mg/mL		
	T47D	IC_50_ = 0.33 mg/mL		
	EMT6/P	IC_50_ = 0.44 mg/mL		
	MDA-MB-231	IC_50_ = 2.13 mg/mL		
Methanolic extract of bran	MCF-7	IC_50_ = 1.29 mg/mL		
	T47D	IC_50_ = 1.01 mg/mL		
	EMT6/P	IC_50_ = 1.19 mg/mL		
	MDA-MB-231	IC_50_ = 2.13 mg/mL		
Water extracted from ground sprouted brown rice powder	A549 (Lung cancer)	IC_50_ = 757.63 μg/mL	Reduction of cancer cell progression through the S phase	[34]
	HepG2	IC_50_ = 732.43 μg/mL		
	KKU-213 (Mixed cholangiocarcinoma)	IC_50_ = 659.23 μg/mL		
Crude ethanolic extract	MDA-MB-231 (Triple-negative breast cancer)	Inhibition = 20% at 100 μg/mL	Anti-invasive activity due to the presence of proanthocyanidins	[35]
	HT1080 (Fibrosarcoma)	Inhibition = 27% at 100 μg/mL		
Hexane fraction	MDA-MB-231	Inhibition = 64% at 100 μg/mL IC_50_ = 50 μg/mL	Decreased secretion and activity of matrix metalloproteinases-2 and -9 and inhibition of collagenase	
	HT1080	Inhibition = 41% at 100 μg/mL		
Dichloromethane fraction	MDA-MB-231	Inhibition = 66% at 100 μg/mL IC_50_ = 45 μg/mL	Collagenase inhibition	
	HT1080	Inhibition = 34% at 100 μg/mL		
Ethanolic extract	Caco-2	IC_50_ = 372.56 μg/mL at 48 h	DNA fragmentation through intrinsic and extrinsic apoptosis pathways	[36]
Aqueous extract		IC_50_ = 3177.60 μg/mL at 48 h		

ND: Not determined.

**Table 2:** In vitro anticancer activity of cereal-derived extracts (maize, wheat, barley, sorghum, oats, and sugar cane) and their mechanisms of action.

Product Used	Cell Line	In Vitro Activity	Mechanism of Action and Other Parameters Evaluated	Reference
Corn (*Zea mays* L.)				
Blue corn extract	HeLa	Inhibition = 61.04% at 1000 μg/mL	ND	[50]
Native corn tortilla extract		Inhibition = 68.69% at 1000 μg/mL		
**Wheat (*Triticum* ssp.)**				
Ethanolic extract of grass	KB (Oral cancer)	IC_50_ = 156 µg/mL	ND	[51]
Methanolic extract of grass	SKBR3 (Breast cancer)	Inhibition = 50% at 600 μg/mL at 48 h	ND	[52]
	SKOV3 (Ovarian cancer)	Inhibition = 48% at 600 μg/mL at 48 h		
Ethanolic extract of the plant	HepG2	IC_50_ = 138.02 µg/mL	Apoptosis	[53]
Aqueous plant extract	Caco-2	IC_50_ = 247.6 µg/mL	Apoptosis	
	HepG2	IC_50_ = 226.4 µg/mL	Apoptosis, increased expression of p53, decreased expression of BCL2, cyclin D1, MMP, and VEGF	
**Barley (*Hordeum vulgare* L.)**				
Water extract	HT-29 (Colorectal adenocarcinoma)	IC_50_ = 0.7 µg/mL	ND	[54]
	A549	IC_50_ = 1.3 µg/mL		
Juice extract	HT-29	IC_50_ = 2.4 µg/mL		
	A549	IC_50_ = 7.4 µg/mL		
**Sorghum (*Sorghum bicolor*)**				
Phenolic extract variety Shawaya short black 1 (with black pericarp)	SW480	Inhibition = ~30%	Activation of apoptosis, p53 expression, and activation of caspases 3 and 7	[55]
Phenolic extract variety IS13116 (brown pericarp)				
**Oatmeal (*Avena sativa* L.)**				
Ethanolic extract of the seed	Caco-2	IC_50_ = 67.0 μg/mL at 24 h and 11.0 μg/mL at 48 h	Apoptosis	[56]
Aqueous extract	MCF-7	Inhibition = 94.3%	ND	[57]
**Sugarcane (*Saccharum officinarum* L.)**				
Ethyl acetate extract fraction	U251 (Glioblastoma)	GI_50_ = 29.1 µg/mL	ND	[58]
	MCF-7	GI_50_ = 26.1 µg/mL		
	NCI-ADR/RES (Multidrug-resistant ovarian cancer)	GI_50_ = 5.9 µg/mL		
	786-0 (Renal cell carcinoma)	GI_50_ = 51.7 µg/mL		
	NCI-H460	GI_50_ = 30.3 µg/mL		
	PC-3 (Ovarian cancer)	GI_50_ = 52.4 µg/mL		
	OVCAR-3	GI_50_ = 35.0 µg/mL		
	HT29	GI_50_ = 34.2 µg/mL		

ND: Not determined.

**Table 3:** In vitro anticancer activity of extracts and nanoparticles derived from vegetables (tomato, eggplant, pepper, and potato) and their mechanisms of action are reported.

Product Used	Cell Line	In Vitro Activity	Mechanism of Action and Other Parameters Evaluated	Reference
Tomato (*Solanum lycopersicum* L. Moench)				
Chloroform extract	AGS (Gastric cancer)	IC_50_ = 55.18 μg/mL	Apoptosis, cell cycle arrest in the G2 phase	[110]
	HCT-116	IC_50_ = 70.73 μg/mL	Apoptosis, decreased percentage of distribution in G0/G1 and increased in G2/M phase	
Zinc oxide nanoparticles from juice	HepG2	Inhibition = ~50% a 50 μg/mL	ND	[111]
Ethanolic extract	HepG2	IC_50_ = 48.0 μg/mL	Apoptosis and DNA damage	[112]
Methanolic Extract	HeLa	IC_50_ = 24.54 μg/mL	Apoptosis	[109]
	H2228 (Lung adenocarcinoma)	Inhibition = ~60–80%	ND	
	H3122 (non-small-cell lung cancer)			
Methanolic extract of plant roots	MCF-7	IC_50_ = 14.34 μg/mL	ND	[108]
	HepG2	IC_50_ = 12.52 μg/mL		
	Caco-2	IC_50_ = 9.69 μg/mL		
Plant root nanocrystals	Caco-2	IC_50_ = 5.73 μg/mL		
	MCF-7	IC_50_ = 20.91 μg/mL		
	HepG2	IC_50_ = 12.08 μg/mL		
**Eggplant (*Solanum melongena* L.)**				
Methanolic extract of the peel	HepG2	IC_50_ = 2.14 μg/mL	ND	[113]
	HCT116	IC_50_ = 4.36 μg/mL		
	HEP2	IC_50_ = 4.99 μg/mL		
	MCF7	IC_50_ = 6.56 μg/mL		
	HELA	IC_50_ = 4.90 μg/mL		
Silver nanoparticles from aqueous extract of fresh leaves	MDA-MB-231	Inhibition = 46.77% at 100 μg/mL	late apoptosis	[114]
Hydroethanolic extract of leaves and branches of the plant	AGS	GI_50_ = 16.7 μg/mL	ND	[101]
	Caco-2	GI_50_ = 11.5 μg/mL		
Ethanolic extract of defatted seeds	T84	IC_50_ = 23.88 μg/mL	Induction of apoptosis mediated by autophagy and PARP1 degradation, altered polymerization/depolymerization of cellular microtubules, and increased LC3β-II expression in T84 cells	[115]
	HCT15 (Colon adenocarcinoma)	IC_50_ = 29.63 μg/mL		
	SF-268 (Glioblastoma)	IC_50_ = 35.36 μg/mL		
	A-172 (Glioblastoma)	IC_50_ = 36.11 μg/mL		
	LN-229 (Glioblastoma)	IC_50_ = 37.11 μg/mL		
	SK-N-SH (Neuroblastoma)	IC_50_ = 37.75 μg/mL		
	PANC-1 (epithelioid carcinoma)	IC_50_ = 49.02 μg/mL		
	MC38	IC_50_ = 39.31 μg/mL		
Ethanolic extract of non-defatted seeds	T84	IC_50_ = 23.88 μg/mL	ND	
	HCT15	IC_50_ = 37.07 μg/mL		
	SF-268	IC_50_ = 23.89 μg/mL		
	A-172	IC_50_ = 24.15 μg/mL		
	LN-229	IC_50_ = 21.22 μg/mL		
	SK-N-SH	IC_50_ = 25.70 μg/mL		
	PANC-1	IC_50_ = 45.29 μg/mL		
	MC38	IC_50_ = 48.58 μg/mL		
**Pepper (*Capsicum annuum* L.)**				
Ethanolic extract of sweet pepper	PC-3	IC_50_ = 78 μg/mL	ND	[116]
Ethanolic extract of chili pepper		IC_50_ = 68 μg/mL		
Crude extract	MCF-7	IC_50_ = 261.97 μM	ND	[117]
	MDA-MB-231	IC_50_ = 241.74 μM		
	K562 (Chronic myeloid leukemia)	IC_50_ = 347.67 μM		
	PANC1 (Melanoma)	IC_50_ = 153.18 μM		
	A375 (Melanoma)	IC_50_ = 393.58 μM		
Aqueous extract of the plant leaves	A549	IC_50_ = 85.45 μg/mL	Ag-Au nanoparticles from aqueous extract of plant leaves induced apoptosis and cell necrosis	[118]
Gold nanoparticles from aqueous extract of plant leaves		IC_50_ = 79.75 μg/mL		
Ag nanoparticles from aqueous extract of plant leaves		IC_50_ = 64.05 μg/mL		
Ag-Au nanoparticles from aqueous extract of plant leaves		IC_50_ = 57.35 μg/mL		
Dry hydroethanolic extract	MDA-MB-231 LoVo (colon cancer)	IC_50_ < 200 µg/mL at 48 h	ND	[119]
**Potato (*Solanum tuberosum* L.)**				
Methanolic extract of plant leaves	MCF-7	IC_50_ = 139.1 μg/mL	ND	[120]
	MDA-MB-231	IC_50_ = 336.4 μg/mL		
	HS578T (Breast cancer)	IC_50_ = 351.6 μg/mL		
Silver nanoparticles from potato peel extract	U118-MG (Malignant brain glioma)	IC_50_ = 6.43 μg/mL	ND	[121]
	Caco-2	IC_50_ = 23.37 μg/mL		
	Skov-3	IC_50_ = 4.31 μg/mL		
Selenium nanoparticles from aqueous extract of potato peel	A549	Inhibition = 50% at 2000 μg/mL at 48 h	ND	[122]
	OVCAR-3	Inhibition = 50% at 2000 μg/mL at 24 h		

ND: Not determined.

**Table 4:** In vitro anticancer activity of extracts and nanoparticles derived from legumes (pea, bean, chickpea, faba bean, lentil, and soybean) and their mechanism of action are reported.

Product Used	Cell Line	In Vitro Activity	Mechanism of Action and Other Parameters Evaluated	Reference
Pea (*Pisum sativum* L.)				
Ethyl acetate extract of peel	MCF-7	Inhibition = 73.6%	Apoptosis, activation of Bax, and a decrease in Bcl-2	[147]
		LC_50_ = 4.31 μg/mL		
Soluble alkaline sulfated extract of peel	HepG2	IC_50_ = 26.9 μg/mL	ND	[148]
Insoluble alkaline sulfated extract of peel		IC_50_ = 28.4 μg/mL		
Acid-insoluble sulfated extract of peel	MCF-7	IC_50_ = 10.6 μg/mL		
Soluble neutral sulfated extract of peel		IC_50_ = 10.3 μg/mL		
Soluble alkaline sulfated extract of peel		IC_50_ = 10.8 μg/mL		
Ethanolic extract of leaves and stems	MCF-7	IC_50_ = 17.67 µg/mL	ND	[149]
Ethanolic extract of husks		IC_50_ = 32.92 µg/mL		
**Beans (*Phaseolus vulgaris* L.)**				
Chloroform extract	MCF-7	Inhibition = 86.44%	ND	[150]
		CC_50_ = 3.84 µg/mL		
**Chickpea (*Cicer arietinum* L.)**				
Sprouted black chickpea extract	MDA-MB-231	IC_50_ = 11.11 µg/mL	ND	[151]
Silver nanoparticles from an aqueous extract of peels	MCF-7	IC_50_ = 82.01 µg/mL	ND	[152]
**Faba beans (*Vicia faba* L.)**				
Nura genotype bean extract (beige color)	AGS	IC_50_ = <0.2 mg/mL	Apoptosis of HL-60 cells	[153]
	HepG2	IC_50_ = <0.2 mg/mL		
	HT-29	IC_50_ = 1.46 mg/mL		
Extract of Rossa genotype faba bean (red color)	AGS	IC_50_ = 1.04 mg/mL		
	HepG2	IC_50_ = 1.59 mg/mL		
	HT-29	IC_50_ = 1.37 mg/mL		
Ethanolic extract	Sk-Mel-28 (Melanoma)	IC_50_ = 118.7 µg/mL	Interference with microtubule organization, causing apoptosis	[154]
Methanolic extract of pods	PC3	Inhibition = 96.57% a 1000 µg/mL	ND	[155]
		IC_50_ = 125.12 µg/mL		
	HepG2	Inhibition = 95.93% a 1000 µg/mL		
		IC_50_ = 126.97 µg/mL		
**Lentil (*Lens culinaris* L.)**				
Lentil extract	MCF-7	IC_50_ = 16.1 µg/mL	Apoptosis	[156]
	HCT-116	IC_50_ = 26.34 µg/mL		
	HepG2	IC_50_ = 45.89 µg/mL		
Silver nanoparticles from lentil extract	MCF-7	IC_50_ = 0.37 µg/mL		
	HCT-116	IC_50_ = 0.35 µg/mL		
	HepG2	IC_50_ = 0.1 µg/mL		
**Soya (*Glycine max* L.)**				
Ethyl acetate fraction of fermented soybeans	HeLa	IC_50_ = 53 µg/mL	Reduction of cell proliferation and induction of G2/M phase arrest, suppression of STAT3 activation through regulation of JNK and Erk1/2 signaling pathways	[157]

ND: Not determined.

## Data Availability

No new data were created or analyzed in this study. Data sharing is not applicable.

## Abbreviations

MTT	3-(4,5-dimethylthiazol-2-yl)-2,5-diphenyltetrazolium bromide
PI3K	Phosphoinositide 3-kinase
IC_50_	Half-Maximal Inhibitory Concentration
ND	Not determined
DPPH	2,2-diphenyl-1-picrylhydrazyl
ABTS	2,2′-azino-bis(3-ethylbenzothiazoline-6-sulfonic acid)
FRAP	Antioxidant Power, Iron Reducing
DNA	Deoxyribonucleic Acid
